# Supplementary figures and images for: Metabolome-Based Discrimination Analysis of Shallot Landraces and Bulb Onion Cultivars Associated with Differences in the Amino Acid and Flavonoid Profiles
Source: Molecules. 2020 Nov 13;25(22):5300. doi: 10.3390/molecules25225300 (PMC7697566; doi:10.3390/molecules25225300)

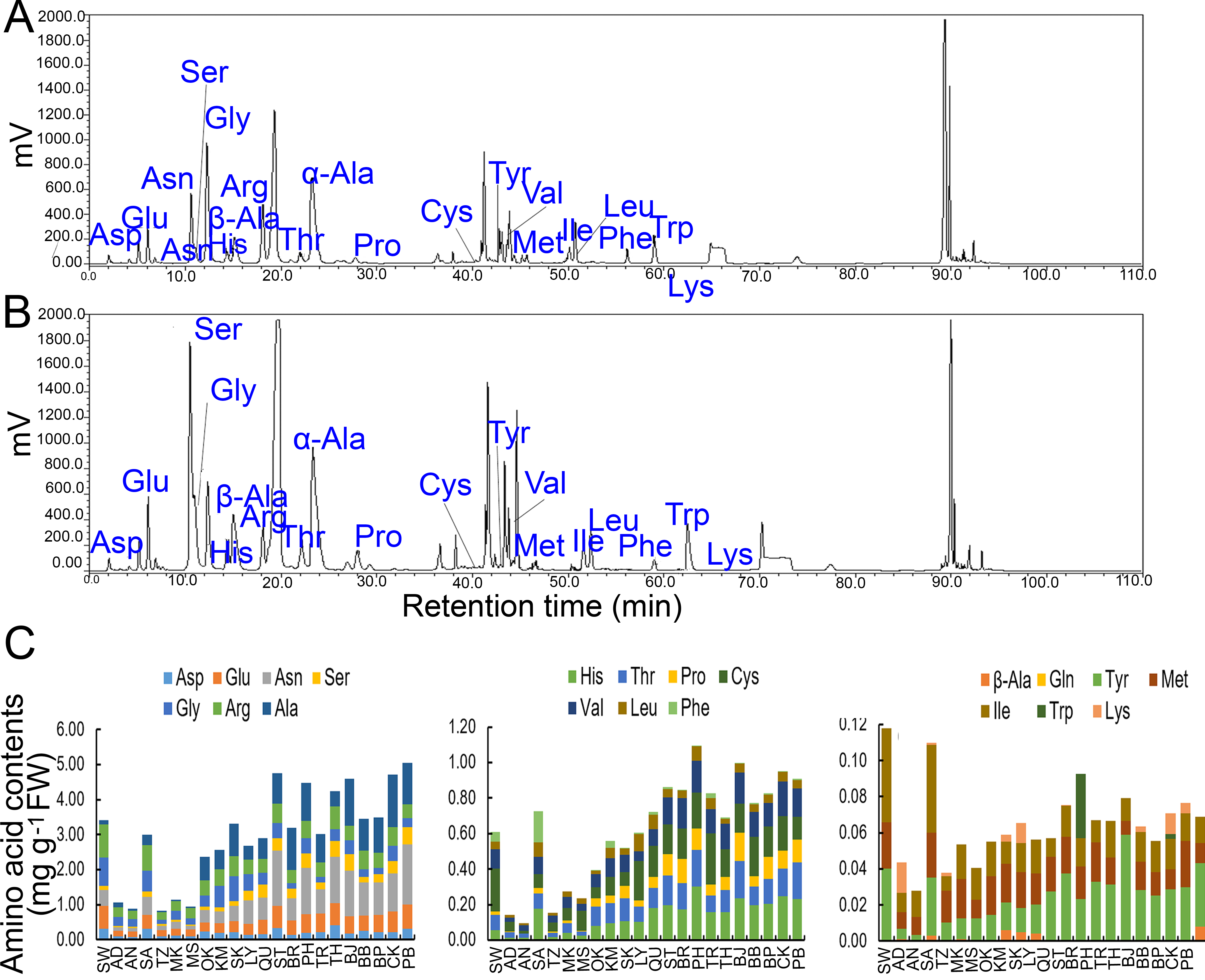

Supplement: Supplementary file 1 [file molecules-25-05300-s001.zip › Figure S1.tif]

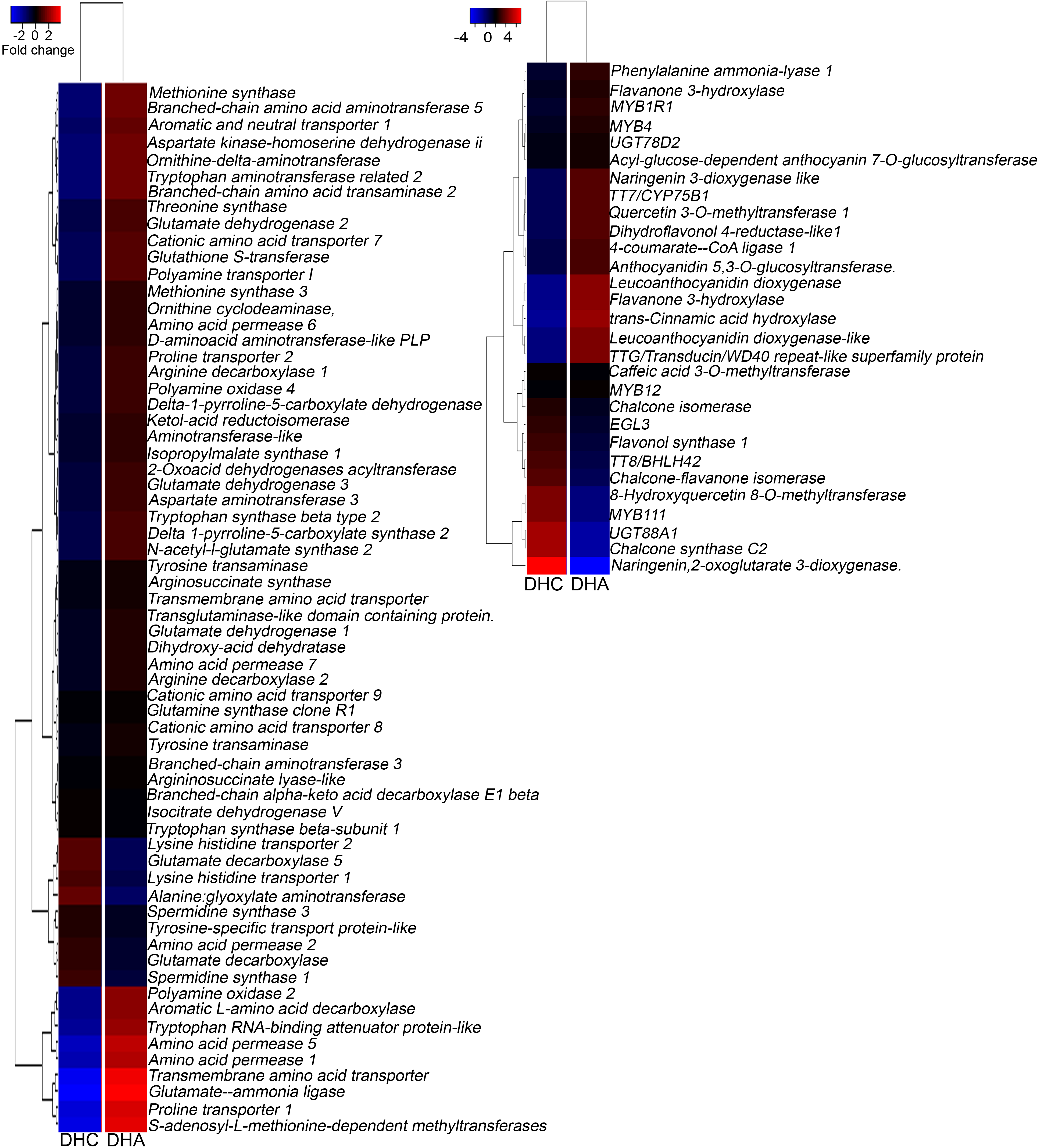

Supplement: Supplementary file 1 [file molecules-25-05300-s001.zip › Figure S2.tif]
